# Evaluation of *Acinetobacter baumannii* infections in intensive care units of a training and research hospital

**DOI:** 10.1186/1753-6561-5-S6-P79

**Published:** 2011-06-29

**Authors:** H Tuzer, AB Besírbellíoglu, S Kílíc

**Affiliations:** 1 Gata, Anakara, Turkey

## Introduction / objectives

The aim of this study was to identify the types of hospital-acquired A. Baumannii infections detected in five intensive care units at GATA Training and Research Hospital between 2008-2010 and to make comparison between years.

## Methods

In this study, patients diagnosed with hospital-acquired infection were evaluated according to the definition by the Center for Disease Control and Prevention (CDC) at a regular surveillance in patients receiving inpatient treatment between 2008-2010 at GATA Training and Research Hospital.

## Results

During this period, patients in five intensive care units were followed up with a regular surveillance. The comparison of the most common types of Acinetobacter Baumannii infections between years revealed that the most common type of infection was blood stream infections with incidence rates of 66.6% in 2008, 72.0% in 2009 and 54.7% in 2010.

## Conclusion

Of all infections detected in the five intensive care units included in this study, the incidence of *Acinetobacter Baumannii* was found to be 17.1% in 2008, 18.8 % in 2009 and 22.6% in 2010. The incidence rate increased over the years in our hospital, which however did not reach statistical significance. (P= 0.437)Concerning the intensive care units, a statistically significant decrease was found in the burn center in the last three years. (P= 0.033) We believe that this decrease results from the decreased number of the inpatients and the education on hand hygiene and isolation precautions provided by the Infection Control Committee during that period.

## Disclosure of interest

None declared.

